# Deoxynivalenol (Vomitoxin)-Induced Anorexia Is Induced by the Release of Intestinal Hormones in Mice

**DOI:** 10.3390/toxins13080512

**Published:** 2021-07-22

**Authors:** Jianming Yue, Dawei Guo, Xiuge Gao, Jiacai Wang, Eugenie Nepovimova, Wenda Wu, Kamil Kuca

**Affiliations:** 1 MOE Joint International Research Laboratory of Animal Health and Food Safety, Engineering Center of Innovative Veterinary Drugs, College of Veterinary Medicine, Nanjing Agricultural University, Nanjing 210095, China; yuejm950322@163.com (J.Y.); gdawei0123@njau.edu.cn (D.G.); vetgao@njau.edu.cn (X.G.); 2 Shandong Vocational Animal Science and Veterinary College, 88 Shengli East Street, Weifang 261061, China; sdmyjcw@163.com; 3 Department of Chemistry, Faculty of Science, University of Hradec Králové, Rokitanského 62, 500 03 Hradec Kralove, Czech Republic; eugenie.nepovimova@uhk.cz; 4 Biomedical Research Center, University Hospital Hradec Kralove, 500 05 Hradec Kralove, Czech Republic

**Keywords:** mycotoxin, trichothecene, deoxynivalenol, intestinal hormones, anorexia

## Abstract

Deoxynivalenol (DON), also known as vomitoxin, is a mycotoxin that can cause antifeeding and vomiting in animals. However, the mechanism of DON inducing anorexia is complicated. Studies have shown that intestinal hormones play a significant part in the anorexia caused by DON. We adopted the “modeling of acute antifeeding in mice” as the basic experimental model, and used two methods of gavage and intraperitoneal injection to explore the effect of intestinal hormones on the antifeedant response induced by DON in mice. We found that 1 and 2.5 mg/kg·bw of DON can acutely induce anorexia and increase the plasma intestinal hormones CCK, PYY, GIP, and GLP-1 in mice within 3 h. Direct injection of exogenous intestinal hormones CCK, PYY, GIP, and GLP-1 can trigger anorexia behavior in mice. Furthermore, the PYY receptor antagonist JNJ-31020028, GLP-1 receptor antagonist Exendin(9-39), CCK receptor antagonist Proglumide, GIP receptor antagonist GIP(3-30)NH_2_ attenuated both intestinal hormone and DON-induced anorectic responses. These results indicate that intestinal hormones play a critical role in the anorexia response induced by DON.

## 1. Introduction

Deoxynivalenol, also known as vomitoxin, is produced mainly by *Fusarium* *graminearum* [1,2]. These *Fusarium* are widely distributed in nature and mainly pollute grains and feeds, such as wheat, barley, and corn [3]. In 1973, Versder first found DON in the feed that caused pigs to vomit in the United States. The WHO also listed DON as one of the food contaminants. After eating food contaminated by DON, humans and animals often have a series of adverse effects, such as anorexia, emesis, weight reduction, changes in neuroendocrine, and immunotoxicity [4,5]. DON is widely distributed and caused a major threat to the health of animals around the world. It has become a research hotspot in veterinary medicine in recent years.

Appetite is usually controlled by central nervous and peripheral factors, which affect the balance of anorexigenic and causative signals in the hypothalamus. In addition to the hypothalamic, the gastrointestinal system is another core of appetite regulation [6]. Enteroendocrine cells (EEC) are located in the gut, accounting for only 1% of the intestinal epithelial cells. Most EECs are open, with microvilli-covered surfaces that are in direct contact with the contents of the intestinal cavity. The enteroendocrine lineage is made up of at least 15 different cell types [7]. The most important function of EEC is to sense intestinal nutrition and respond by secreting a sequence of peptides and amine hormones, especially intestinal hormones. Intestinal hormones can act as neurotransmitters and circulating hormones, and connect the signals of nutrients in the alimentary tract with a sequence of physiological reactions through the brain–gut axis [8]. Intestinal antifeedant hormones that affect appetite include cholecystokinin (CCK), glucagon-like peptide 1 (GLP-1), Peptide YY (PYY), and gastric inhibitory peptide (GIP) [9,10,11,12].

Both PYY and GLP-1 are secreted by enteroendocrine L cells, where cell density gradually increases from the proximal to the distal end of the intestine [13]. PYY belongs to the pancreatic polypeptide family. As an important satiety hormone, PYY can upregulate the anorexic signaling molecules in many species, such as primates and rodents [14,15]. Studies have shown that PYY mainly binds to the NPY2 receptor in the brain [16]. Only when PYY binds to the Y2 receptor at the terminal of the vagus afferent nerve can it transmit anorectic signals to the brain to inhibit neuropeptide Y (NPY) and activate opioid melanin Proto (POMC), which causes antifeeding reaction. GLP-1 was first discovered as an incretin, which can prevent hyperglycemia by enhancing insulin secretion and inhibiting the secretion of glucagon [17]. GLP-1 also induce satiety signals through the vagus nerve, or reduce gastric emptying and intestinal peristalsis to influence the appetite [18].

GIP is a linear peptide composed of 42 amino acids, which belongs to the incretin hormone family, and released from enteroendocrine K cells in the duodenum and jejunum. GIP plays a significant role in the animals by inhibiting gastric acid secretion and gastric motility, stimulating the secretion of insulin and glucagon, and regulating postprandial glucose homeostasis [19]. As a brain-gut peptide, CCK can be secreted in the intestinal endocrine cells, and is also widely distributed in the central and peripheral nervous system. Injecting CCK into the middle of the pons and hypothalamus can cause obvious food refusal, which is one of the evidences that CCK is involved in central appetite regulation as a neurotransmitter [20]. Studies have found that CCK and PYY play important roles in the anorectic response induced by DON [21,22,23].

The purpose of this research is to verify the tentative that DON attracts the secretion of intestinal hormones from enteroendocrine cells to cause mice anorexia, and to compare the difference between DON intragastric administration and intraperitoneal injection. We use the established “modeling of acute refusal in mice” as the experimental model, and pretreatment with intestinal hormone receptor antagonists. We found gut hormone CCK, PYY, GIP, and GLP-1 can lead to poor feeding behavior in mice, while gut hormone receptor antagonists can inhibit the anorectic reaction induced by DON. These results indicate that DON may act on the intestinal endocrine cells to induce intestinal antifeedant hormones.

## 2. Results

### 2.1. DON Induces Acute Anorectic Reaction in Mice

**Figure 1:**
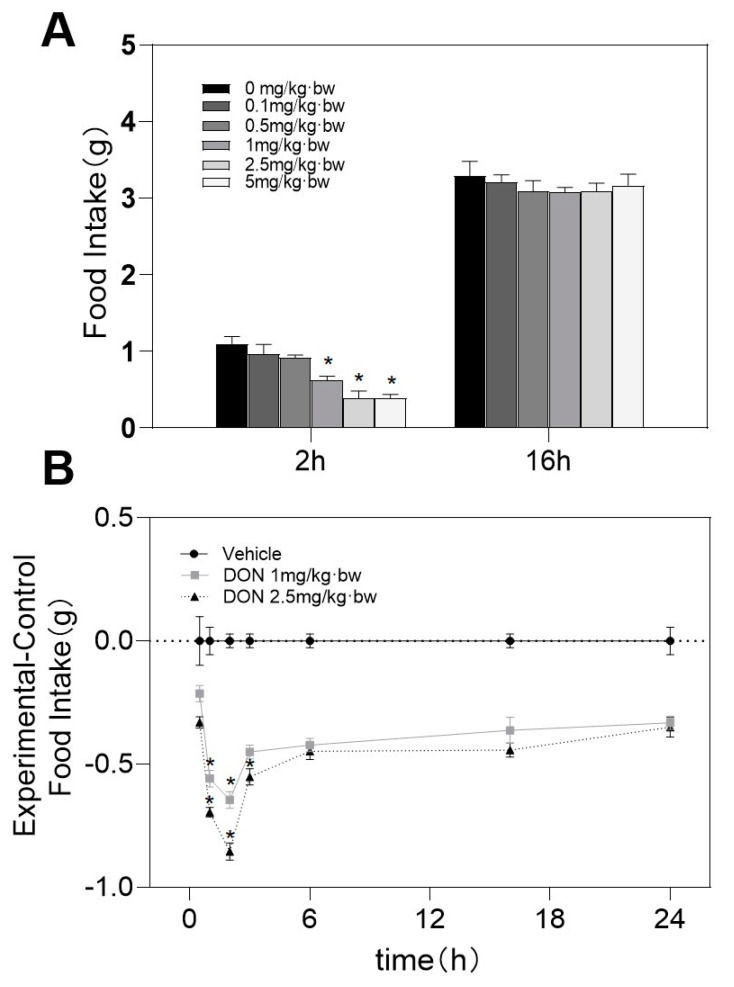
(**A**) Response of feed intake dose and (**B**) kinetic of antifeedant induced by DON gavage. (**A**) Mice were gavaged with 0, 0.1, 0.5, 1, 2.5, and 5 mg/kg bw toxins. We used the Holm–Sidak method to perform one-way ANOVA, to analyze the significance of multiple groups. (**B**) Mice were gavaged with PBS, 1 or 2.5 mg/kg bw toxins. We used the Holm–Sidak method to perform two-way repeated ANOVA (one factor) to analyze significant differences compared with the control group. Data are expressed in the form of mean ± SEM (n = 6). Symbols: * indicates difference in DON groups compared with the control group (*p* < 0.05).

**Figure 2:**
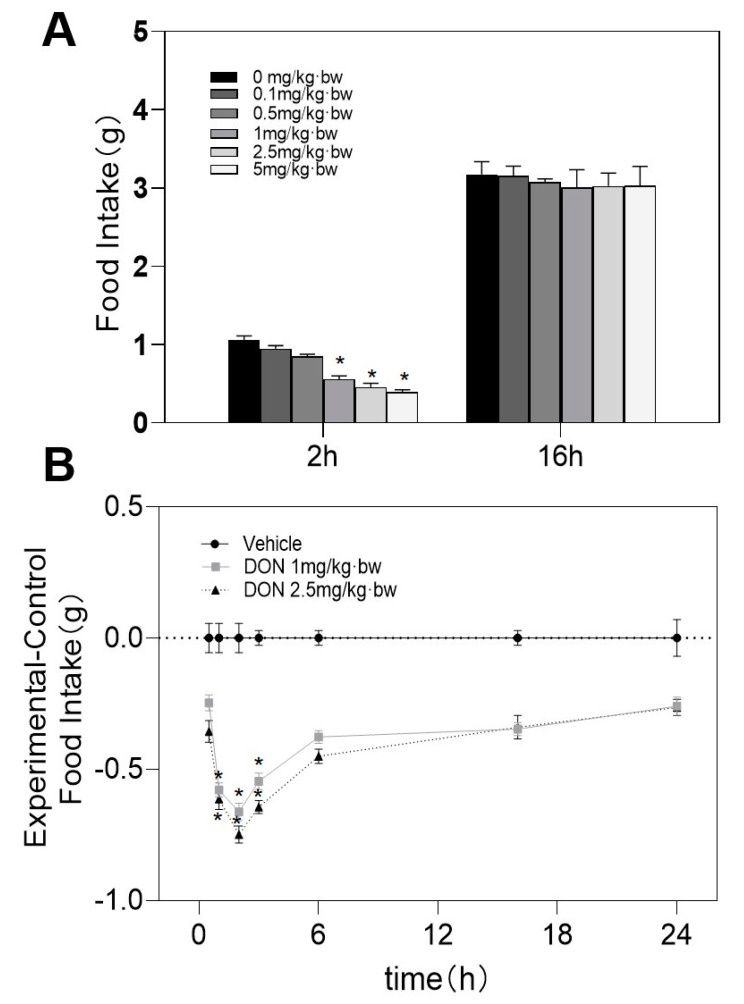
(**A**)Response of feed intake dose and (**B**) kinetic of antifeedant induced by intraperitoneal injection of DON. As shown in the Figure 1 legend, experiments were carried out and the data were evaluated. * indicates difference in DON groups compared with the control group (*p* < 0.05).

After 2 h of intragastric administration or intraperitoneal injection of DON, 1, 2.5, and 5 mg/kg·bw toxins showed a decrease in feed intake. Although the feed intake of mice in the 0.1 and 0.5 mg/kg·bw groups decreased, they were no differences being observed between control and toxin group. After 16 h, they were not significantly different from the control group (Figure 1A and Figure 2A). However, being exposed to 5 mg/kg bw DON, the mice showed symptoms of arched backs and frizzy hair. So we gave up 5 mg/kg·bw and chose 1 or 2.5 mg/kg·bw DON groups for further research on the anorectic response. Within 3 h after the DON challenge, the feed intake dose of 1 and 2.5 mg/kg·bw groups decreased significantly in contrast to the control group (Figure 1B and Figure 2B).

### 2.2. DON Evokes Plasma Intestinal Hormones Elevation

**Figure 3:**
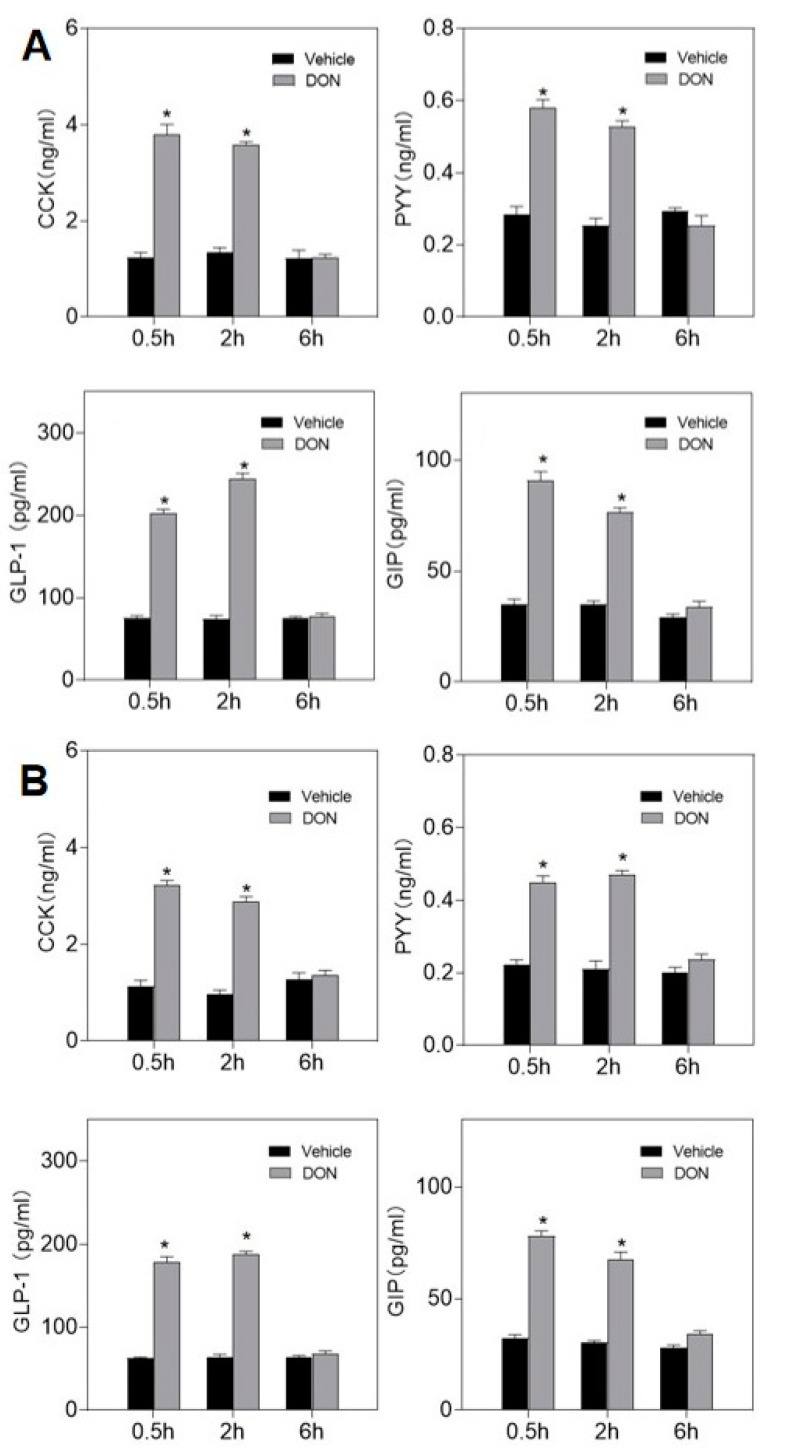
Dose responses of DON-induced CCK, PYY, GLP-1 and GIP concentrations in plasma. (**A**) Mice were gavaged with 2.5 mg/kg bw DON. (**B**) Mice were intraperitoneally injected with 2.5 mg/kg bw DON. Data are expressed in the form of mean ± SEM (n = 6). Holm–Sidak was used to perform one-way ANOVA to analyze the significance of multiple groups. Symbols: * indicates difference in DON groups compared with the control group (*p* < 0.05).

Mice were gavaged and intraperitoneally injected with 2.5 mg/kg·bw DON, and the results were consistent with the feed intake experiment. The concentrations of hormones CCK, PYY, GIP, and GLP-1 in plasma at 0.5 and 2 h significantly exceeded the control, but all of them returned to normal levels at 6 h (Figure 3). Mice challenged by gavage secreted higher levels of hormones than mice intraperitoneally injected.

### 2.3. DON and Intestinal Hormone-Induced Anorexia Is Reduced by Intestinal Hormone Receptor Antagonists

**Figure 4:**
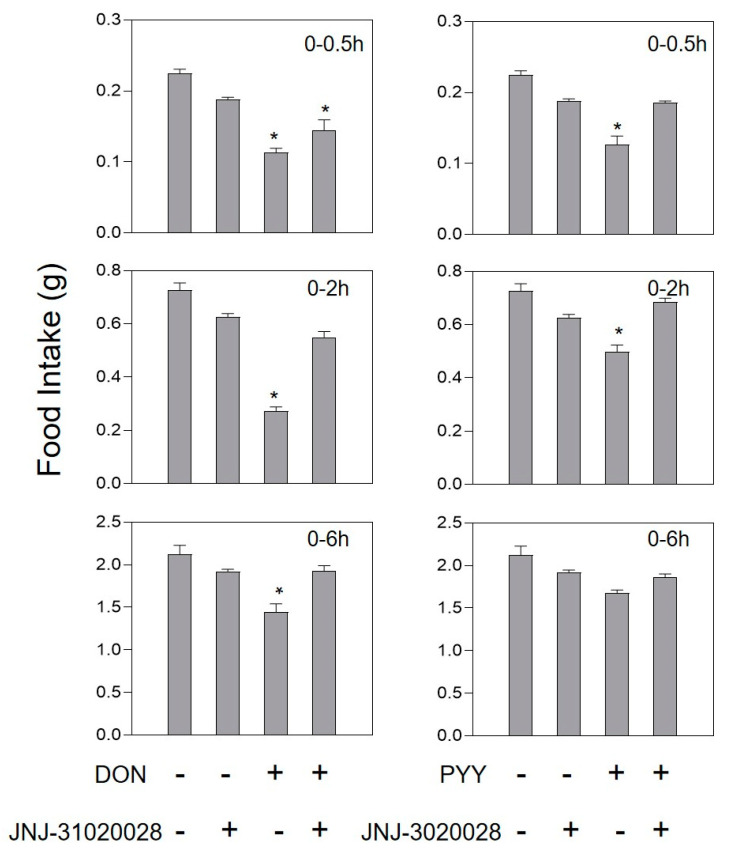
Effect of JNJ-31020028 on anorexia caused by PYY or DON. Mice were preprocessed with 10 mg/kg·bw JNJ-31020028 or PBS. After 30 min, mice were challenged with 0.1 mg/kg·bw PYY or 2.5 mg/kg·bw toxin. Data are expressed in the form of mean ± SEM (n = 6). Holm–Sidak was used to perform two-way repeated ANOVA (one factor) to analyze significant differences compared with the control group. Symbols: * indicates a significant difference compared with the control (*p* < 0.05).

**Figure 5:**
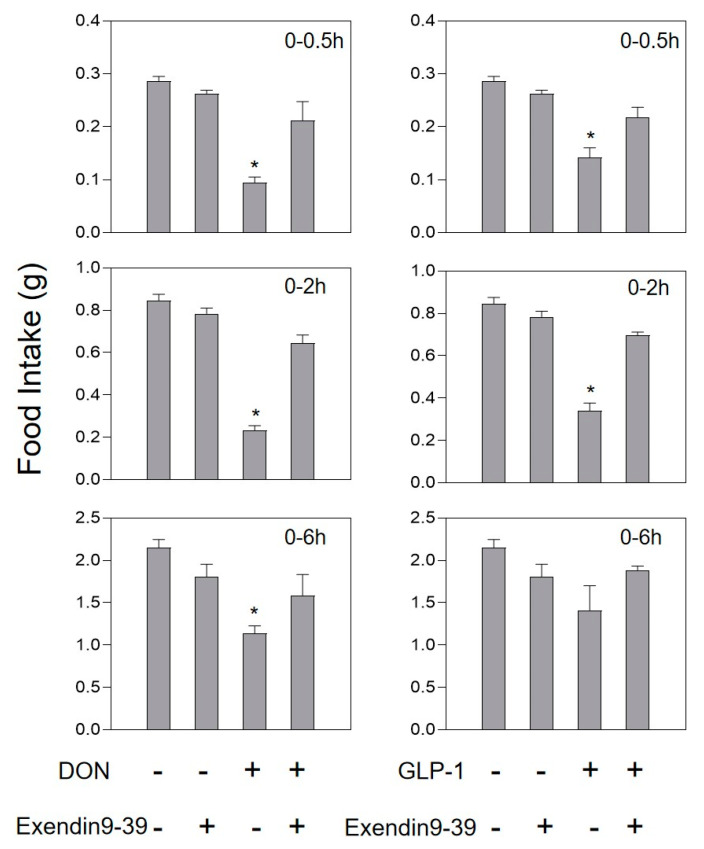
Effect of Exendin(9-39) on anorexia caused by GLP-1 or DON. Mice were preprocessed with 0.1 mg/kg·bw Exendin(9-39) or PBS. After 30 min, mice were challenged with 0.01 mg/kg·bw GLP-1 or 2.5 mg/kg·bw toxin. As shown in the Figure 4 legend, experiments were carried out and the data were evaluated. * indicates difference in DON groups compared with the control group (*p* < 0.05).

Compared with the control group, mice injected with 0.1 mg/kg·bw PYY or GLP-1 could significantly reduce feed intake at 0.5 and 2 h, and then return to normal after 6 h. The intestinal hormone receptor antagonists Exendin9-39 and JNJ-31020028 had no effect on food intake dose in mice compared with the control group (Figure 4 and Figure 5).

After pretreatment of 10 mg/kg·bw JNJ-31020028, at 0.5 h, the mice had 35.4% more dose of food intake than DON groups. After 2 h, there were no significant differences compared to the control group (Figure 4). When the GLP-1R antagonist Exendin(9-39) was pretreated, the feed intake of GLP-1-treated mice did not change compared with the control group. When Exendin(9-39) and DON worked together, the feed intake of mice returned to normal levels within 0.5 h (Figure 5).

**Figure 6:**
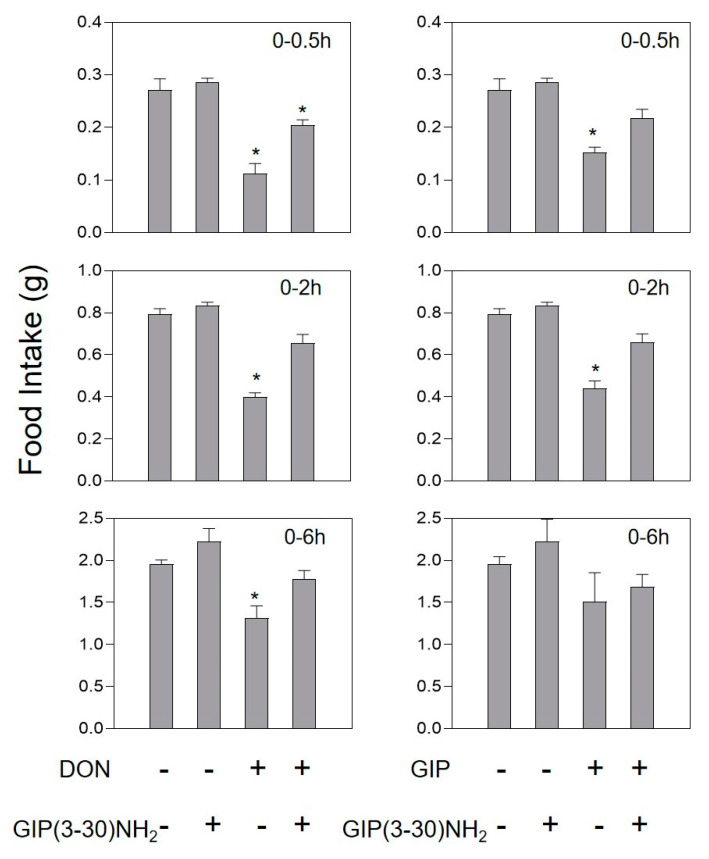
Effect of GIP(3-30)NH2 on anorexia caused by GIP or DON. Mice were intravenous injection of 50 nm/kg·bw GIP(3-30)NH2 or PBS. After 30 min, mice were challenged with 0.1 mg/kg·bw GIP or 2.5 mg/kg·bw DON. As shown in the Figure 4 legend, experiments were carried out and the data were evaluated.* indicates difference in DON groups compared with the control group (*p* < 0.05).

**Figure 7:**
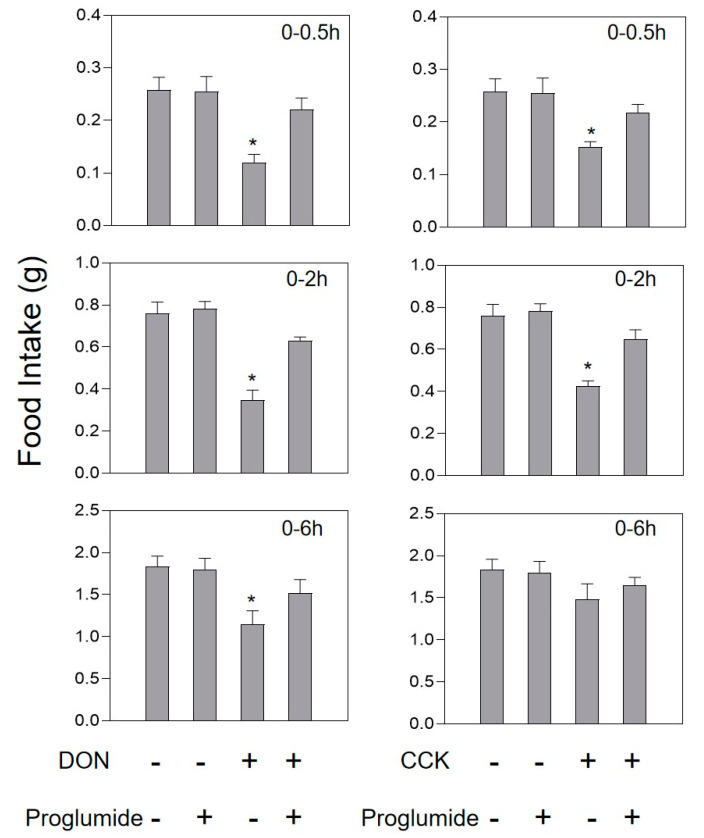
Effect of Proglumide on anorexia caused by CCK or DON. Mice were preprocessed with 50 nm/kg·bw Proglumide or PBS. After 30 min, mice were challenged with 0.1mg/kg·bw CCK or 2.5mg/kg·bw toxin. As shown in the Figure 4 legend, experiments were carried out and the data were evaluated.* indicates difference in DON groups compared with the control group (*p* < 0.05).

Intraperitoneal injection of 0.1 mg/kg·bw GIP and 0.01 mg/kg·bw CCK in mice could significantly reduce feed intake at 0.5 and 2 h, and return to normal levels after 6 h (Figure 6 and Figure 7). After intravenous injection of the GIP receptor antagonist GIP(3-30)NH_2_ alone, the feed intake of mice increased, but with no significant difference (*p* > 0.05). However, when GIP(3-30)NH_2_ and DON acted together, the food intake of the mice was reduced by 29.4% compared to the control in 0.5 h, and the food intake of the mice was back to normal after 2 h (Figure 6). There was no significant difference in the feed intake dose of the mice after single intragastric administration of 400 mg/kg·bw CCK-A/B receptor antagonist Proglumide. Pretreatment of mice with Proglumide could completely inhibit the anorexia caused by DON (Figure 7).

## 3. Discussion

From a public health perspective, the biggest problem with DON is that it can cause anorexia and affect the growth of young animals, but the underlying mechanism of the clinical response of this toxin is still poorly understood. In this study, we focused on the effect of intestinal satiety hormone in anorexia caused by DON. Our findings here demonstrate that DON rapidly induces four intestinal hormones CCK, GLP-1, GIP, and PYY in the plasma of mice; the way this increase occurs is consistent with the induction of anorexia. Additionally, the observed DON-induced addition in intestinal hormones was inhibited by the intestinal hormone receptor antagonist. These experiments confirmed that intestinal satiety hormones play an important role in DON-induced anorexic response.

The established “modeling method for acute anorexia in mice” was used as the basic experimental model to design [23]. The results of anorexia induced by DON are highly consistent with previous research results [24]. DON can acutely induce anorexia in mice within 3 h, and it is concentration-dependent. After 3 h, the mice’s food intake returns to normal. When mice are exposed to DON, the reduction in food intake within the first 3 h may initiate a compensation mechanism, and increase food intake dose within 3–16 h. When we gavaged mice with 5 mg/kg·bw DON, we discovered that some mice showed symptoms of arched backs and frizzy hair, but the feed intake returned to normal at 6 h, which proved that high doses of DON may cause pathological damage to mice. This may be related to the high-dose DON induced expression of pro-inflammatory genes in mice [25].

DON can also induce the concentration of mouse plasma intestinal satiety hormones GLP-1, GIP, PYY, and CCK to rise rapidly within 2 h, while the intestinal anorectic hormone in mouse plasma returns to normal levels after 6 h, which is consistent with the anorexia before. In addition, these satiety hormones can be stimulated by other trichothecenes [21,23]. Moreover, we found that the effect of inducing hormone secretion by DON gavage challenge is more significant than that of intraperitoneal injection, which may be related to the site where DON induces anorexia. The intraperitoneal injection of DON is absorbed by the omentum into the blood circulation, while the intragastrically injected DON directly acts on the gastrointestinal tract through the digestive system, with faster action and higher concentration. Studies have shown that DON can act on mouse enteroendocrine cells (STC-1) to cause the release of CCK and GLP-1 [26]. Therefore, we speculate that the main site of DON-induced anorexic response in mice is the epithelium of the intestinal lumen. DON interacts with intestinal endocrine cells to induce the release of intestinal hormones, to control the central and peripheral anorexia and feeding signal regulation. However, how DON interacts with receptors on the surface of intestinal endocrine cells still needs further exploration.

As an important satiety hormone, PYY can induce anorexia by upregulating anorexia signaling molecules [14,15]. Studies have shown that PYY is a peptide that could penetrated the blood–brain barrier and bind to the NPY2 receptor in the brain to transmit anti-feeding signals to the brain [16]. Here, PYY administered by intraperitoneal injection caused mice to refuse to eat, indicating that this hormone might be the cause of the DON-induced anorexia. We improved the previous experiment [22] and used the NPY2 receptor antagonist JNJ-31020028 instead of BIIE0246. Because only 2% of BIIE0246 could cross the blood–brain barrier in mice, it has little effect on DON-induced anorexia [27]. As a specific NPY2 receptor antagonist, JNJ-31020028 can penetrate the blood–brain barrier [28]. Therefore, it has the ability to block Y2R in the central and peripheral areas, and can comprehensively explore the role of NPY2 receptor and PYY in DON-induced anorexia. As demonstrated here, administrations of PYY and DON elicited anorexia, and JNJ-31020028 could attenuate anorexic response, indicating that NPY2R plays an important part in DON-induced anorexia.

CCK works through two different receptor subtypes (CCK-A and CCK-B). CCK-A is located in the entire gastrointestinal tract and central nervous system, and plays a vital role in CCK-induced satiety [29]. CCK-B is expressed and integrates signals of pain, angst, and memory in the brain. In addition to the brain, CCK-B can also be expressed in gastrointestinal tissues, vagus nerve afferent fibers, and adrenal glands [30]. It is worth noting that, in previous mice research [22], Flannery found that mice developed anorexia after intraperitoneal injection of CCK in mice, which is consistent with our results. Although it has been shown that intraperitoneally injecting the CCK1R antagonist devazepide can reverse the anorexia caused by DON, but the difference is not statistically significant. In subsequent experiments, we delivered the CCK1R antagonist SR 27897 to the mice by oral gavage, which significantly reduced the anorexia caused by DON and CCK [23]. This time we used Proglumide, a non-peptide and orally active CCK-A/B receptor antagonist [31], to gavage mice to selectively block the effect of CCK in the central nervous system. In our experiments, Proglumide indeed suppressed both CCK- and DON-induced anorexia. Therefore, compared with intraperitoneal injection, oral CCK receptor antagonists may be more effective in obtaining the concentration required to inhibit CCK in DON-driven feed refusal.

GIP activates the GIP receptor (GIPR) in the pancreatic islets, to enhance glucose-stimulated insulin secretion. GIP receptor is a G protein-coupled receptor and is expressed in many organs, including the gastrointestinal tract, adipose tissue, and central nervous system [32]. In 2016, a new GIPR antagonist GIP(3-30)NH_2_ was discovered for the first time [33]. GIP(3-30)NH_2_, as the N- and C-terminal truncated GIP peptide, has a strong specific GIPR antagonistic effect, and can play an antagonistic effect in mice. In addition, GLP-1 induces satiety signals through the vagus nerve, or reduces appetite by reducing gastric emptying and bowel movement, which is adjusted by the CNS-induced c-fos immunoreactivity [17,18]. Moreover, GLP-1R also existed in the intestine and is an important binding site for GLP-1. We observed that the GIP receptor antagonist GIP(3-30)NH_2_ can inhibit anorexia caused by exogenous GIP or DON, and the anorexia response induced by GLP-1 and DON can be effectively controlled by the GLP-1R antagonist Exendin(9-39) inhibition, which is consistent with previous studies [21]. However, further experiments are needed to clarify the specific molecular mechanism of DON in the peripheral release of intestinal satiety hormone.

## 4. Conclusions

In summary, this study found that DON can acutely induce anorexia in mice within 1–3 h, and it can also acutely increase the concentration of intestinal hormones CCK, PYY, GIP, and GLP-1 in mice plasma. We also proved that the intestinal hormones CCK, PYY, GIP, and GLP-1 can trigger anorexia behavior in mice, and the receptors of these hormones play an essential role in the antifeedant response induced by DON. These results indicate that intestinal satiety hormones and receptors play a critical role in the antifeedant response induced by DON. However, the molecular mechanism related to this effect still needs to be studied in the future.

## 5. Materials and Methods

### 5.1. Animals

We purchased 10-week female B6C3F1 mice from the Comparative Medicine Center of Yangzhou University. Each mouse was housed individually in a cage, maintained at a temperature of 21–24 °C, a relative humidity of 40-55%, and cycled under 12 h of light/darkness. The mice were fed a high-fat diet (45% fat content; Jiangsu Medicine Company, China).

### 5.2. Toxins and Chemicals

DON (purity >98%) was obtained from Sigma-Aldrich (St. Louis, MA, USA). DON was dissolved in sterile phosphate buffered saline (PBS) containing 1% dimethyl sulfoxide (DMSO) and dissolved to 0.1, 0.5, 1, 2.5, and 5 mg/kg·bw. GIP (MedChemExpress; Trenton, NJ, USA), PYY, and CCK (Sigma-Aldrich; St. Louis, MO, USA) were respectively diluted in PBS, at a dose of 0.1 mg/kg·bw. GLP-1 (MedChemExpress) was made into a 0.01 mg/kg·bw solution with PBS. The PYY receptor antagonist JNJ-31020028, the CCK receptor antagonist Proglumide (Sigma-Aldrich), and GLP-1 receptor antagonist Exendin(9-39) were respectively dissolved in 100 μL sterile PBS, so that their final doses were 10, 200, or 0.1 mg/kg·bw. GIP receptor inhibition GIP(3-30)NH_2_ was synthesized by the Syn peptide (Shanghai, China), diluted with normal saline to 50 nm/kg·bw, and injected through the tail vein. According to the instructions of the reagent supplier, previous animal research and thesis data, the dosages of various pharmacological agents were selected [27,31,34,35].

### 5.3. DON Induces Acute Anorexia in Mice

The general experimental design on anorexia and toxins in mice is based on previous experiments in the laboratory [23]. The mice were randomly grouped according to their body weight, with 6 mice in each group, which were fasted from 10:00 a.m. to 6:00 p.m. Mice were orally gavaged or injected intraperitoneally with 0.1, 0.5, 1, 2.5, and 5 mg/kg·bw DON. At 2 and 16 h after exposure, the dose of food intake was measured. To evaluate the kinetics of anorexia induced by DON, mice were orally gavaged or intraperitoneally injected with 100 μL of 1 mg/kg·bw toxin; we measured food intake at 0.5, 1, 2, 3, 6, 16, and 24 h after exposure.

### 5.4. DON-Induced Plasma Hormone Measurement

Mice were respective orally gavaged or intraperitoneal injected with 0 or 2.5 mg/kg·bw DON. At 0, 0.5, 2, and 6 h after exposure, the mice were euthanized with ether, the abdominal cavity was immediately opened to expose the inferior vena cava, and blood was collected with anticoagulant tubes. The plasma was centrifuged at 3500 x g for 10 min at 4°C to separate the serum and snap frozen at −80 °C. Using CCK, GLP-1, PYY, GIP enzyme immunoassay kit (Phoenix Pharmaceuticals; Burlingame, CA, USA) to measure plasma hormone levels and reading the plate absorbance at 450 nm by an ELISA plate reader.

### 5.5. Effect of Antagonists on Anorexia Caused by Hormones or DON

The general experimental design on anorexia and toxins in mice is based on previous experiments in the laboratory [22]. The first group of mice was injected with 50 μL of PBS and intestinal hormones; the second group of mice was injected with intestinal hormone receptor antagonists and intestinal hormones; the third group of mice was injected with PBS 30 min before the test, and 2.5 mg/kg·bw DON during the test; the fourth group was injected with an intestinal hormone receptor antagonist and 2.5 mg/kg·bw toxin; the fifth group of mice was injected with intestinal hormone receptor antagonist and 50 μL PBS; the sixth group of mice were injected intraperitoneally with 50 μL PBS 30 min before and during the test. The experiment was applied to PYY and its receptor antagonist JNJ-31020028, GLP-1 and its receptor antagonist Exendin(9-39), CCK, and its receptor antagonist Proglumide and GIP, and its receptor antagonist GIP (3-30)NH_2_. The food intake of the mice was then measured 0.5, 1, and 2 h later.

### 5.6. Statistics

We used Sigma Plot 11.0 (Jandel Scientific; San Rafael, CA, USA) for data analysis, and when *p* < 0.05, the difference was significant. We used the Holm–Sidak method to perform one-way ANOVA to analyze significance of multiple groups. We used the Holm–Sidak method to perform two-way repeated ANOVA (one factor) to analyze significant differences in food intake doses and the concentrations of intestinal hormones CCK, PYY, GIP, and GLP-1 in comparison to the control group at various times.

## Data Availability

The data presented in this study are available upon request to the corresponding author.
